# Prevention in practice: why is it neglected and what can we do?

**DOI:** 10.3399/bjgp22X719429

**Published:** 2022-04-29

**Authors:** Paul Aveyard, Susan Jebb

**Affiliations:** Nuffield Department of Primary Care Health Sciences, University of Oxford, Oxford; National Institute for Health Research (NIHR) Oxford Biomedical Research Centre, Oxford University Hospitals Trust, Oxford.; Nuffield Department of Primary Care Health Sciences, University of Oxford, Oxford; NIHR Oxford Biomedical Research Centre, Oxford University Hospitals Trust, Oxford.

*‘If the moon, in the act of completing its eternal way around the earth, were gifted with self-consciousness, it would feel thoroughly convinced that it was traveling its way of its own accord on the strength of a resolution taken once and for all. So would a Being, endowed with higher insight and more perfect intelligence, watching man and his doings, smile about man’s illusion that he was acting according to his own free will.’*

(Albert Einstein)

## THE PROBLEM

In the UK, one in seven adults smoke and more than one in four have obesity. Clinicians universally recognise these conditions as important preventable causes of early morbidity and mortality. Clinical consultations allow clinicians to intervene opportunistically on these risk factors but intervention is uncommon, even when patients consult with disease caused by the risk factor.^1^ This is irrational, judged by explicit medical standards embodied by guidelines: opportunistic behavioural interventions are effective and cost-saving,^2^ something rarely true for other medical treatment.

When a preventive interaction occurs, clinicians typically advise people to change behaviour rather than offer support to achieve this. Despite expanding a pay-for-performance system to incentivise opportunistic support for smoking cessation, intervention frequency did not increase, and advice was given 30 times more frequently than support.^3^ Similarly, advice on obesity rather than referral to support is clinicians’ most common intervention.^1^ Qualitative evidence shows that patients report receiving banal and insulting advice from doctors, but welcome support when offered.^4^

## CLINICIANS’ EXPLANATIONS

Researchers have asked clinicians to account for their failure to follow preventive guidelines. Common explanations are that it takes too long, it is ineffective, and it could offend the patient.^5^ These appear to be rationalisations, not reasons. A study that received these standard explanations for non-engagement found that, through observation, the real issues lay with what physicians valued and understood as their roles.^6^ Even direct experience that brief opportunistic interventions can be quick, effective, and well-received does not displace these rationalisations.^7^ In a focus group study, we asked clinicians to explain the reasons for the difference in their approach to obesity and hypertension. The only explanation was that hypertension was treated by pills, suggesting that physicians lack an underpinning philosophy of what defines what they do and do not value, and thereby how they practise.

## THE PRESTIGE HIERARCHY

Sociological observation of medicine suggests we value specialties that are *‘active, specialized, biomedical, and highly-technological … practiced on organs in the upper part of the bodies of young or middle-aged people’*.^8^ Neurosurgery and cardiology are prized; public health and psychiatry are at the bottom of the hierarchy. We value diseases similarly, and this plays out in physicians’ attitudes towards patients with these conditions.^9^^,^^10^ Our papers are commonly rejected from prestigious medical journals with a suggestion that they are more suitable for specialist journals. Yet the contents list of leading general medical journals suggests that these journals could commonly be mistaken for specialist oncology journals: the prestige hierarchy at work.

## THE ROLE OF STIGMA IN PHYSICIANS’ BEHAVIOUR

Freidson argued that high-prestige diseases are serious, legitimate, and not stigmatising.^11^ Smoking and obesity do not compromise normal life in the immediate term, they do not exempt one from normal obligations and confer the sick role, and they are considered shameful. We suggest they are shaming because our implicit mental model is that people choose to smoke and choose to overconsume food. Even education about the genetic and neurobiological basis of these behaviours appears not to change our perception that behaviour is chosen and that change mostly requires one to really want to change.^12^ We perceive that only intrinsic motivation and willpower will be effective or appropriate.

## THE DECEPTION OF CONSCIOUSNESS

Our mental model of free choice reflects what our interior life feels like. If I am free to choose, so must you be, and your choices become stigmatising because you choose to smoke or overeat despite knowing the harms. However, our sense of rational choice is not supported by evidence. A study examined the behaviour of Israeli judges deciding for or against granting parole.^13^ Immediately after a break, judges were likely to grant parole and rarely granted it immediately before a break, suggesting mood influenced their decisions. This study affirms that inner drives, not rational choosing, are key factors, not only of unconscious behaviour, but also of deliberative acts in trained professionals exercising a deliberative role. The lesson lies in how judges might account for their decisions. Judges would be unlikely to perceive that their judgements reflected their inner drives more than facts. Rather, for every case, there are reasons to be lenient and reasons to be harsh, and judges’ attention to, and weighing of, these elements was influenced by their drive state without their awareness.

More generally, we can perceive some inputs to our decisions and we can know the choice we make, but the process of weighing itself is opaque. Our consciousness interpolates this as rational choosing, but we cannot know whether it is or not. This is analogous to walking through a busy city, where we can navigate without bumping into people and still recognise our friend in the crowd. We cannot perceive how the brain integrates inputs, only that it does so by perceiving the output.^14^ We feel we are the ‘ghost in the machine’, the conscious chooser in Einstein’s quote, when genetics, neuroscience, and observations of behaviour tell us we are not. Our mental model of choosing helps explain clinicians’ behaviour on prevention. If the patient can choose, what is my role? If I have told the patient what they should do, what more can I do?

## TWO NEW SUGGESTIONS TO ADDRESS THE UNDERLYING PROBLEMS

Guideline implementation strategies for preventive behavioural interventions typically educate physicians on their importance, show that the barriers physicians report do not apply, and train physicians in how to intervene. This has limited impact. We suggest instead that implementation strategies could be improved by inviting physicians to consciously scrutinise the value system within medicine. We would struggle to defend values embodied by the prestige hierarchy to each other and to patients, and they are at odds with the expressed values of healthcare systems. That said, the values tacitly revealed by clinical norms reflect and are reinforced by society. Television coverage of the COVID-19 pandemic was dominated by younger people on intensive care units, when the main burden of disease lay elsewhere, in older people dying alone. Publicly examining our implicit value system may create distaste towards it that could overcome our current distaste for preventive care.

Likewise, implementation strategies might benefit from making the deception that Einstein verbalised — that we are free conscious choosers — fit with our everyday experience of apparently exercising free will. Understanding neurobiology has typically not driven out mind–body dualism and the notion of free will. This is where ‘stories’, such as the Israeli judges study, can illuminate. It is a human story with which we can empathise and might prompt reflection on our own confidence as clinicians in the ‘choices’ we make in medicine. Understanding and applying science to benefit patients is the essence of medicine — the answer to the medical school interview question. Preventive care may be low tech, but it needs clinicians to engage with relevant science and evidence every bit as much as personalised treatment for cancer. Doing so may allow us to feel more empathy with those whose ‘choices’ might appear to discredit them. Our patients will benefit, and we will know ourselves better.
